# Treatment of AC dislocation by reconstructing CC and AC ligaments with allogenic tendons compared with hook plates

**DOI:** 10.1186/s13018-018-0879-x

**Published:** 2018-07-11

**Authors:** Guheng Wang, Renguo Xie, Tian Mao, Shuguo Xing

**Affiliations:** 1 Department of Hand Surgery, Affiliated Hospital of Nantong University, 20# West Temple Road, Nantong, 226001 People’s Republic of China; 2 Department of Hand Surgery, Shanghai General Hospital, 650# Songjiang Road, Shanghai, 201620 People’s Republic of China

**Keywords:** Surgery, Acromioclavicular joint dislocation, Coracoclavicular ligaments, Acromioclavicular ligaments, Allogenic tendon, Hook plate

## Abstract

**Background:**

The purpose of this study was to compare outcomes between allograft reconstruction and hook plate fixation for acute dislocation of the acromioclavicular joint with a minimum 2-year follow-up.

**Methods:**

A retrospective comparative study of patients treated for acute acromioclavicular joint dislocation from February 2010 to December 2014 in our hospital, consisting of 16 patients who were followed-up, was performed.

Eight patients were treated for acute AC dislocation and underwent surgical reconstruction as follows: the coracoclavicular and acromioclavicular ligaments were reconstructed with the allogenic tendon. The other eight patients were treated with hook plates to maintain the AC joint reset. At the latest follow-up, radiographic analysis and the Constant and University of California-Los Angeles (UCLA) scores were used to evaluate shoulder function. The satisfaction of the patients in terms of the efficacy and visual analog scale (VAS) data were also recorded.

**Results:**

After an average follow-up of 30.3 months (range 24–46 months), no patient had dislocated their joint again at the final follow-up based on X-ray examination. The Constant score was 94.4 for the allogenic tendon group and 93.8 for the hook plate group (*P* = 0.57). According to the UCLA scale (*P* = 0.23) or VAS (*P* = 0.16), we found no significant difference between the two groups. All patients reported that they were very satisfied or satisfied with the outcome of surgery, and no significant difference (*P* = 0.08) was found between the two groups.

**Conclusions:**

The use of allogenic tendon for reconstruction of the coracoclavicular and acromioclavicular ligaments shows excellent outcomes in terms of the recovery of clinical function or radiographic outcomes for acute AC dislocation. Compared with the hook plate, the hardware did not need to be removed.

## Background

Acromioclavicular (AC) joint dislocation is a common injury, which accounts for approximately 9% of shoulder injuries [1]. When AC joint dislocation occurs, it not only produces shoulder pain and abnormal activity symptoms but also greatly affects the strength, flexibility, and movement of the entire upper extremity. Because previous techniques are associated with frequent complications, such as loss of reduction, fracture of the coracoids, and loosening [2–6], many studies have evaluated potential improvements in the surgical management of AC joint dislocation. Many methods exist, indicating that an ideal method still needs to be explored. Considering the possibility of vertical and anteroposterior displacement of the clavicle in AC joint dislocation, we adopted a method to reduce and maintain the reduction of the AC joint using an allogenic tendon to reconstruct the coracoclavicular and acromioclavicular ligaments in acute injuries. We also compared it with the clavicular hook plate treatment to assess the merits and demerits of this method.

## Methods

### Patients

A retrospective study of patients with acute AC joint dislocation who were treated in our hospital between February 2010 and December 2014 was performed. The institutional ethics committee approved the study, and informed consent was obtained from all study participants. In this study, eight patients (six male, two female) with an average age of 49.0 years were treated with an allogenic tendon to reconstruct the coracoclavicular and acromioclavicular ligaments after AC joint dislocation. At the same time, eight atients (five male, three female) with an average age of 41.3 years were treated with the hook plate for fixation of AC joint dislocation. Patients with chronic dislocation (≥ 3 weeks after trauma) and patients who received any operative treatment of the injured shoulder were excluded. Patients with accompanying coracoid fracture, shoulder wounds, and chronic infections were also excluded from this study.

According to the Rockwood classification, there were six cases of type III, two of type IV, and eight of type V. The mean time from injury to surgery was 2.9 (range 1–5) days. Of all injuries, 14 were caused by traffic accidents, 1 by a simple fall, and 1 by blunt trauma (Table 1).

**Table 1:** Data of the 16 evaluated patients

Patient	Age/sex side	Mechanism of injury	Plate removal (months)	Follow-up (months)	Type of separation^a^
Allogenic tendon					
1	34/male right	Fall from height	–	28	IV
2	43/male left	Traffic accident	–	43	V
3	59/male left	Traffic accident	–	24	V
4	64/female right	Traffic accident	–	29	IV
5	72/male left	Traffic accident	–	29	III
6	23/male left	Traffic accident	–	33	III
7	34/female right	Traffic accident	–	27	V
8	63/male left	Traffic accident	–	25	V
Hook plate					
1	56/male right	Blunt trauma	10	30	III
2	22/male left	Traffic accident	7	31	III
3	34/male left	Traffic accident	5	24	V
4	31/male left	Traffic accident	8	26	V
5	42/female right	Traffic accident	11	46	III
6	37/female right	Traffic accident	8	39	III
7	63/male right	Traffic accident	11	25	V
8	45/female left	Traffic accident	12	26	V

^a^According to the Rockwood classification [29]

### Surgical technique

#### Allogenic tendon

**Fig. 1:**
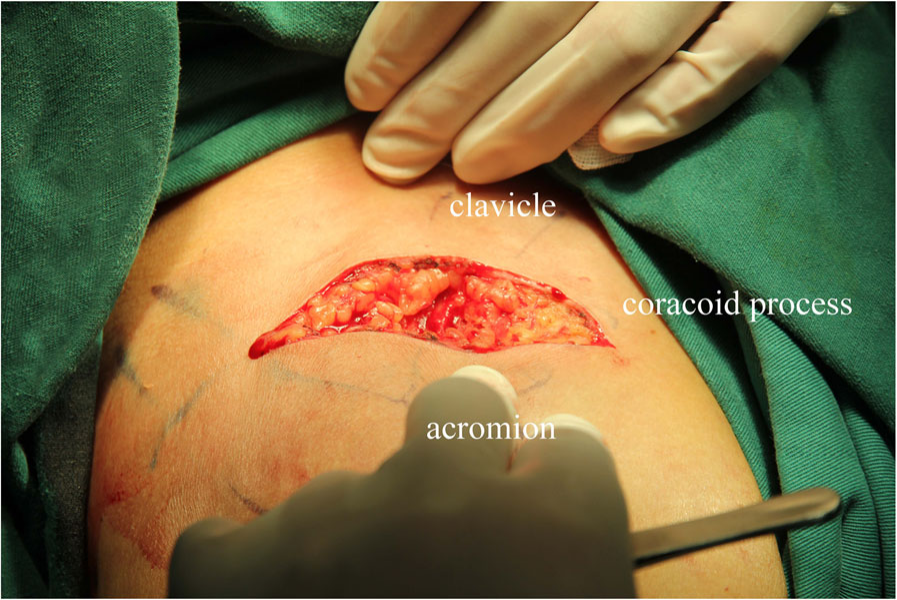
Saber cut incision spanning the AC joint to just proximal to the coracoid process

Under interscalene regional block or general anesthesia, the patient was placed in the beach-chair position and the upper limb draped free. A saber cut incision (Fig. 1) was made in line from the coracoid process to the medial AC joint. After development of subcutaneous flaps, the deltotrapezial fascia was taken down subperiosteally, exposing the clavicle, AC joint, and coracoid process [7].

**Fig. 2:**
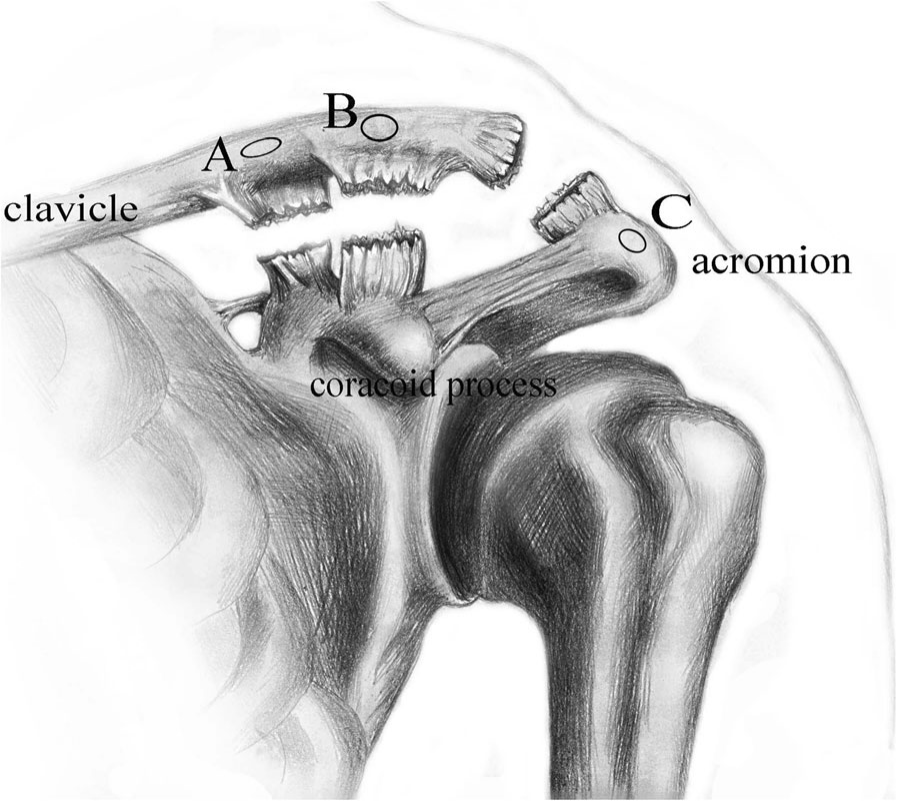
Diagram of intra-operative drilling position. **a** Tunnel: approximately 40 mm from the distal end at the clavicle using a 3.5-mm drill bit. **b** Tunnel: approximately 20 mm from the distal end at the clavicle using a 4.0-mm drill bit. **c** Tunnel: approximately 15 mm to the AC joint at the acromion using a 3.5-mm drill bit

After the AC joint was exposed, the coracoclavicular (CC) and AC ligaments were identified to ensure that they were ruptured. With the deltoid flap retracted, the base of the coracoid process was exposed, and a soft tissue tunnel was made. After the AC joint was reduced, the reduction was maintained with direct pressure with assistance. Corresponding to the trapezoid ligament and conoid ligament attachment in the clavicle, two tunnels, using a 4.0 and a 3.5 mm drill bit, were drilled separately at a distance of approximately 20 and 40 mm from the distal end of the clavicle (Fig. 2a, b). Another drill tunnel (3.5 mm) was positioned on the acromion approximately 15 mm from the AC joint (Fig. 2c). This corresponds to the acromioclavicular ligament. At this time, the allogenic tendon (Tissuebank of the Orthopedic Institute of the People’s Liberation Army in Beijing) was taken out of the sealed bag, washed with saline, and placed in saline with antibiotics for 30 min before use. The flexor digitorum profundus tendon was usually selected because its length and width meet the requirements. (The time from tendon harvest to use was approximately 1–2 months.) Tendon donors were usually healthy and died between 20 and 50 years old. Their deaths were often caused by accidents. If one tendon was not long enough, we used two allogenic tendons to reconstruct the coracoclavicular ligament and the acromioclavicular ligament separately. Attention was paid to ensure that the graft looked firm and appeared fresh.

**Fig. 3:**
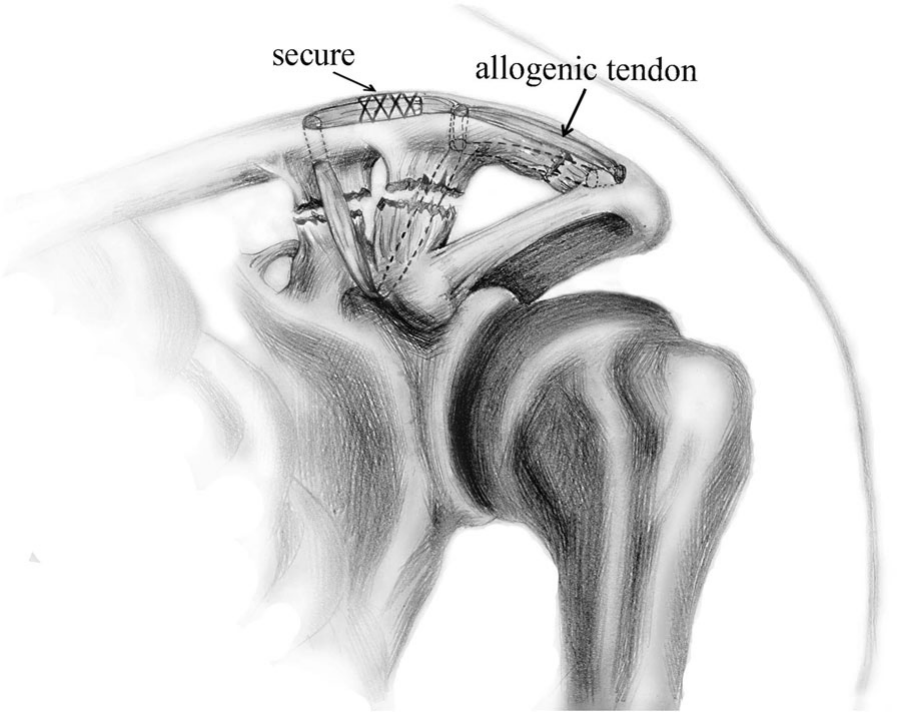
Diagram of allogenic tendon used to reconstruct the coracoclavicular and acromioclavicular ligaments

**Fig. 4:**
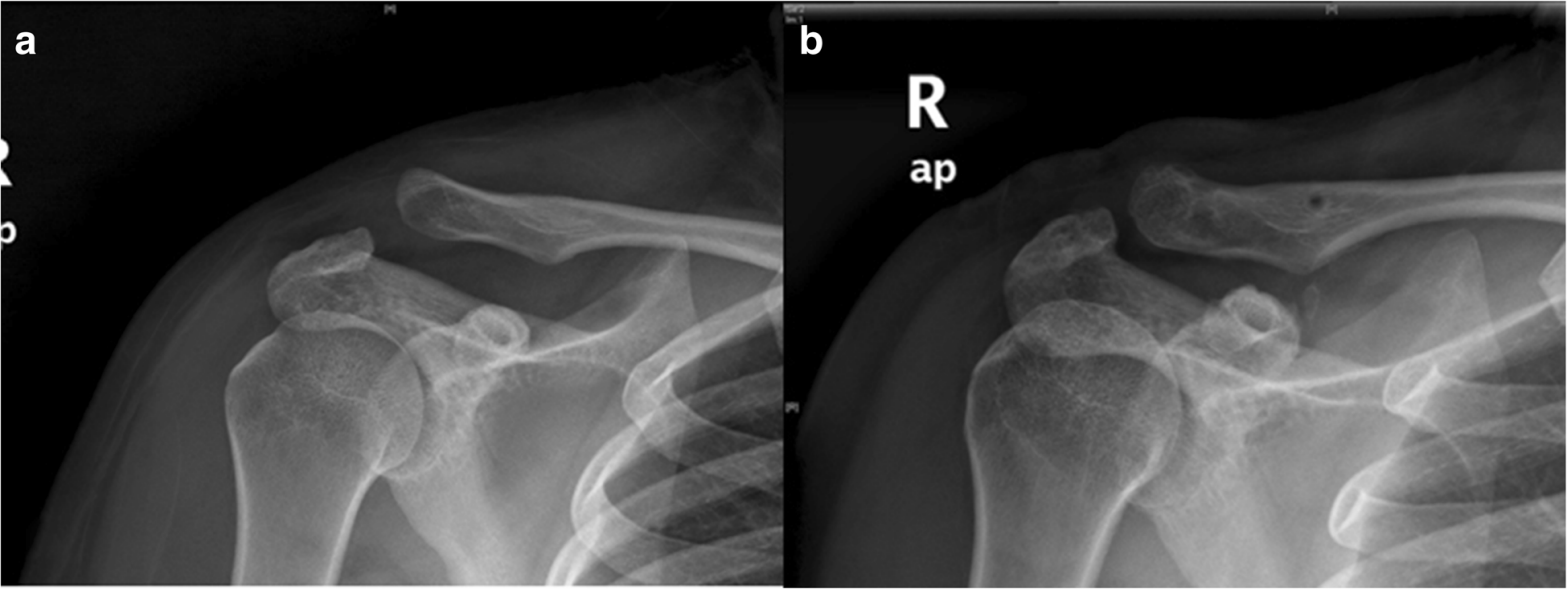
**a** X-rays of AC joint dislocation before surgery. **b** X-rays of AC joint dislocation after surgery reconstructing the coracoclavicular and acromioclavicular ligaments with the allogenic tendon

The prepared allogenic tendon was then pushed through the A tunnel with a curved hemostat. Next, the allogenic tendon was passed around the coracoid process and then, with the help of a passing wire, passed through the B tunnel, which was previously made. Then, the allogenic tendon was continually passed through the C tunnel at the acromion on the clavicle. Finally, the allogenic tendon was pushed through the B tunnel again below the clavicle to reach the surface of the clavicle. Subsequently, the two free ends of the allogenic tendon were secured to each other on the surface of the clavicle with maximum manual tension between the A and B tunnels using 3-0 nonabsorbable surgical suture (Ethibond Johnson & Johnson, New Brunswick, US) (Fig. 3). Throughout the process, assistance helped to maintain the reduction of the AC joint, and both the trapezoid and conoid ligaments as well as the acromioclavicular ligament were reconstructed. Next, the trapezius-deltoid fascia was repaired and the wound closed. Before and after surgery, X-rays of the AC joint were obtained, and the dislocation is shown in Fig. 4. The X-ray after surgery confirmed that the AC joint was restored.

#### Hook plate

The surgical position and anesthesia were performed as in the allogenic tendon group. Centered on the AC joint, a 5- to 7-cm skin incision was made to expose the dislocation and ensure that the coracoclavicular and acromioclavicular ligaments were ruptured. The articular disc was removed if injured. The hook plate was then used to fix the dislocation (3.5 mm LCP clavicular hook plate, Synthes GmbH, Solothurn, Switzerland) as the end of the hook was inserted under the acromion. Before application of the hook plate, the depth of the acromion was measured, and the plate was pre-bent to perfectly fit the clavicle. When the hook plate was placed, 3.5-mm screws were used to fix it in place. The coracoclavicular and acromioclavicular ligaments were then repaired with #5 nonabsorbable surgical suture (Ethibond Johnson & Johnson, New Brunswick, US). Finally, the deltoid trapezoid fascia was closed with resorbable sutures, and the wound was closed in layers. The hook plate was removed at 9.0 (range 5–12) months after the surgery.

### Postoperative management

A sandbag was used to compress the wound for 24 h after surgery. Postoperatively, the patient was placed in a shoulder immobilizer for 4 weeks, although movement of the wrist and elbow was encouraged immediately after surgery. After 4 weeks, shoulder joint motion, including the pendulum exercise, began; however, heavy physical work was not permitted until 3 months. Patients were not allowed to participate in sports activities until 6 months after the operation.

### Clinical evaluation

All patients were asked to report whether they have any particular discomfort in the shoulder, which may be related to the overall surgery satisfaction (very satisfied, satisfied, partially satisfied, or not satisfied).

During follow-up, the appearance of the shoulder was assessed to determine whether there were any deformities, such as projections of the distal clavicle. At the same time, we also took X-rays and evaluated the radiographic findings, including the occurrence of osteoarthritic changes and the complete reduction degree of the AC joint. A visual analog scale (VAS: range 0–10; 0 represents no pain and 10 represents maximal imaginable pain) was used to evaluate the pain postoperatively.

Clinical evaluation of patients was performed using both the Constant [8] and University of California-Los Angeles (UCLA) [9] scoring systems. The Constant score is graded from 0 to 100, where 100 is best possible score, and consists of four dimensions: pain (0–15 points), activity level (0–20 points), range of movement (0–40 points), and power (0–25 points). We assumed that scores ≥ 90, 80–89, 70–79 and < 70 indicated excellent, good, fair, and poor, respectively. The UCLA score consisted of pain (0–10 points), function (0–10 points), range of motion (0–5 points), strength (0–5 points), and the patient’s satisfaction (0–5 points). The total UCLA score is 35 points, and 34 or 35, 29–33, and ≤ 29 indicated excellent, good, and poor, respectively.

### Statistical methods

The *t* test was used to evaluate significant differences between the two study groups for continuous variables, and the Wilcoxon test was used to evaluate significant differences for patient satisfaction during follow-up. A value of *P* less than 0.05 was considered as statistically significant. Statistical analysis was performed with SPSS version 16.0 software (SPSS, Chicago IL, USA).

## Results

In the allogenic tendon group, the operative procedure was performed by the corresponding author, whereas all patients in the hook plate group were operated on by seniors. No patient reported any immune problems related to the allogenic tendon or hook plate.

In the allogenic tendon group, the eight patients were followed up, and the total follow-up ranged from 25 to 43 months, with a mean of 29.8 months. In the hook plate group, the total follow-up ranged from 24 to 46 months, with a mean of 30.9 months. No patients lost reduction on the final follow-up based on the X-ray results, and no patients had shoulder deformities.

All patients reported that they were very satisfied or satisfied with the outcome of the surgery. Three patients were satisfied, and five were very satisfied in the allogenic tendon group, whereas two patients were satisfied and six were very satisfied in the hook plate group. Although, there were more “very satisfied” results in the hook plate group, this difference was not significant (*P* = 0.08).

All patients returned to work without pain, and the VAS scale showed no significant difference between the two groups (*P* = 0.16).

### Function

No significant differences were found between the two groups with regard to the Constant score (*P* = 0.57), with 94.4 in the allogenic tendon group (86–100, number in brackets indicates the range of the variables) compared to 93.8 in the hook plate group (84–98) (Table 2). The results for all patients were rated as excellent or good according to the score. Detailed results of the functional questionnaire are shown in Table 2.

**Table 2:** Clinical functional outcome of treated shoulder

	Pain (15)	Activity level (20)	Range of movement (40)	Power (25)	Constant score (100)
Allogenic tendon	14.3	19.5	37.3	23.4	94.4
Hook plate	14.4	19.4	37.8	22.3	93.8
*P* value	> 0.05	> 0.05	> 0.05	> 0.05	> 0.05

No significant differences were found between the two groups with regard to the UCLA score, with 33.5 (30–35) for the allogenic tendon group and 34.1 (31–35) for the hook plate group (*P* = 0.23).

## Discussion

The aim of our study was to introduce and evaluate the method using allogenic tendon to reconstruct the CC and AC ligaments for acute AC joint dislocation, and we also compared it with the hook plate to determine its merits and disadvantages. We believe that Rockwood types IV and V of AC joint injuries require operative treatment, and we proposed surgical treatment for patients with type III lesions involved in heavy labor or patients who participate in sports activities.

The tendon allograft played an important role in tendon and ligament reconstruction, particularly for the patients who have a shortage of autograft tendons or who do not want to use their own normally functioning tendons. Allograft tissue has many advantages over autograft tissue, including unlimited size, lack of donor site morbidity, and availability for revision surgery. The drawbacks of allogeneic tendons are that they may cause minimal immunogenicity and increase the chance of rejection compared with autograft tissues. The tendon as an allograft has low cellularity and is preserved at − 80° to elicit minimal immunogenicity. In our study, we did not find any adverse reactions after grafting of allogenic tendons, and the preliminary clinical assessment did not find any significant adverse reactions [10–12]. Currently, the risk of viral transmission through allograft tissue transplantation is extremely low due to proper donor screening and tissue processing. The tendons we used were fully tested to exclude infectious diseases and sterilized to prevent the risk of infection from grafts. This documentation allowed us to use tendons from this company for recipients in our country. Therefore, we consider that reconstructing the CC and AC ligaments with allogenic tendons is a safe approach.

Up to now, more than 70 types of methods have been described for the treatment of acute AC joint dislocation, but no one method has been considered the best [13–32]. These surgical techniques can be grouped broadly into types such as fixation of the AC joint or fixation between the coracoid and clavicle and as dynamic muscle transfer or ligament reconstruction. The current literature shows the conoid ligament guarding against anterosuperior loading and the trapezoid guarding against posterior loading of the clavicle [33]. Debski et al. also clarified that the AC ligaments play an important role in constraint of horizontal motion of the distal clavicle [34]. Predicting that reconstructing the CC and AC ligaments with allogenic tendon would provide good stability of the AC joint, we designed and used this method and compared it with the hook plate procedure.

In the anatomic reconstruction method, two clavicle tunnels were used, and the allogenic tendon was passed around the base of the coracoid process (V shape), which recreated the anatomy of both the conoid and trapezoid ligaments. Based on the advantages of the V-shape technique, it was more likely that the patient would achieve greater overall stability by reducing the amount of abnormal translation. To successfully reconstruct the conoid and trapezoid ligaments using this technique, adequate exposure to the base of the coracoid is helpful and should be obtained. Subcoracoid suture placement is not completely anatomical, and poor visualization risks injury to nearby neurovascular structures; therefore, the separation and drilling should be performed with extreme care to maintain coracoid integrity and avoid potential fractures. The method we used was similar to that of Saccomanno [35] and was used at nearly the same time. The difference is that we used allogenic tendons and omitted the process of harvesting tendons.

There are several causes of chronic pain after the surgical treatment of AC dislocations, one of which is persistent anteroposterior instability of the clavicle [36, 37]. By adding AC ligament reconstruction, the horizontal stability of the clavicle will be further strengthened. In our study, the VAS was 0.38, indicating that the results are quite good. Biomechanical studies of AC joint reconstruction with free-tissue graft for both the CC and AC ligaments provide AC joint stability similar to that of the intact AC joint and significantly better than that of the modified Weaver-Dunn procedure [38, 39]. Carofino and Mazzocca [40] used a technique that involves reconstruction of the superior AC ligament and capsule. In the presented method, we recreate the superior and inferior AC ligament and attain better stability. The results are encouraging and satisfactory.

Because of the good clinical outcomes, the hook plate remains one of the most commonly used methods for acute AC dislocations [41–44]. According to follow-up, the results confirmed that the efficacy of the clavicular hook plate was high. The advantage of the hook plate was the relatively easy implantation procedure and that it can provide immediate stability, which allows rapid healing of the torn ligaments and ensures early rehabilitation with a minimal risk of loss of reduction or implant failure. However, the implant had to be removed, which was a disadvantage and increased the rate of separation and medical costs. Compared with the hook plate, the method of allogenic tendon reconstruction did not require a second surgery and reduced the patient’s pain.

We did not find any infections or other complications in either series. The hook plate is a reliable fixation tool for complete AC joint dislocations, ensuring immediate stability and allowing early mobilization with good functional and cosmetic results. With allogenic tendon grafts, the damaged anatomy can be restored without sacrificing any tendons or ligaments. Based on several in vitro biomechanical studies [38, 45–48] and our research, allogenic tendon graft reconstructions are likely to provide available alternatives for the treatment of operable AC joint dislocations. By controlling both the vertical and anteroposterior displacements of the clavicle, this technique offers strong biological reconstruction to maintain the AC joint reduction. Limitations of our study include the small number of patients and only acute injuries being studied. Further studies are necessary to confirm the reliability of this new method.

## Conclusions

We prefer allografts because there is no donor site morbidity involved, and they are of adequate length to loop around the coracoid and over the clavicle. Moreover, they are readily available at our institution. Both techniques, allogenic tendon reconstruction and hook plate fixation, are effective procedures for the surgical treatment of AC joint acute dislocations of Rockwood III, IV, and V. There is no principle difference in functional outcomes between the two treatments; however, patients with allogenic tendon reconstruction do not require hardware removal and have less pain.

## Abbreviations

AC: Acromioclavicular
CC: Coracoclavicular
UCLA: University of California-Los Angeles
VAS: Visual analog scale
